# Seasonal Change in Adiponectin Associated with Ovarian Morphology and Function in Wild Ground Squirrels (*Citellus dauricus* Brandt)

**DOI:** 10.3390/ijms232314698

**Published:** 2022-11-24

**Authors:** Sijie Fan, Wenjing Lu, Haolin Zhang, Zhengrong Yuan, Yingying Han, Qiang Weng

**Affiliations:** College of Biological Science and Technology, Beijing Forestry University, Beijing 100083, China

**Keywords:** adiponectin, adiponectin receptor, MAPK signaling pathway, ovary, steroidogenesis, wild ground squirrels

## Abstract

The goal of this study is to explore the relationship between altered circulating adiponectin concentration, ovarian tissue morphology, ovarian steroidogenesis, and sex hormone production in ovaries of wild ground squirrels. The ovarian mass differed significantly during the breeding and non-breeding seasons, and the circulating estradiol and progesterone concentrations were significantly higher in the breeding season, while the circulating adiponectin level was significantly lower. The expression levels of gonadotropin receptors (FSHR and LHR) and steroidogenic enzymes (StAR, P450scc, P450arom, and 3β-HSD) were significantly higher during the breeding season. Comparing the ovarian transcriptome data of wild ground squirrels between the two periods, we found that some differentially expressed genes were enriched for ovarian steroidogenesis and the adipocytokine signaling pathway, which correlated with our present results. Notably, the MAPK signaling pathway was also enriched and its related genes (*Erk1*, *p38 Mapk*, *Jnk*) were up-regulated by qPCR during the non-breeding season. These findings suggested that adiponectin may be involved in the regulation of seasonal changes in the ovarian function of wild ground squirrels, possibly by acting on the MAPK signaling pathway to regulate sex steroidogenesis in the ovaries.

## 1. Introduction

Adiponectin, a 30 kDa peptide hormone, is the most abundant protein secreted by white adipose tissue. Adiponectin monomers undergo processes, such as post-translational modification, to form multiple multimers and are highly expressed in the circulation of rodents and humans [1]. The isoforms of adiponectin include low-molecular-weight (LMW) trimers, medium-molecular-weight (MMW) hexamers, and high-molecular-weight (HMW) octadecamers. HMW adiponectin appears to be the most biologically active, accounting for half of the total adiponectin in serum [2]. As a major regulator of insulin sensitivity, adiponectin plays an important role in regulating energy homeostasis [3]. In the organism, adiponectin exerts physiological effects by binding to adiponectin receptors (AdipoR1, AdipoR2, and T-cadherin), which are widely distributed in tissues and organs [4]. There is substantial evidence that adiponectin inhibits type II diabetes, obesity, atherosclerosis, and inflammation [5,6,7]. In recent years, increasing attention has been paid to the effects of adiponectin on the reproductive system, especially in females. Interestingly, adiponectin levels show sexual dimorphism, with plasma HMW adiponectin concentrations significantly higher in females than males, suggesting a possible link between adiponectin and sex steroid synthesis and secretion [8]. In addition, other isoforms of adiponectin may be involved in reproductive regulation, and abundant LMW adiponectin was detected in female follicular fluid [9]. Adiponectin deficiency may cause impaired follicular development, disturbed ovarian steroidogenesis, and the development of ovarian-related disorders, including polycystic ovary syndrome (PCOS) [10]. The expression of AdipoR1 and AdipoR2 has been reported in mammalian ovarian somatic cells and follicles, suggesting that adiponectin may directly act on the ovary to regulate follicle development and steroidogenesis [11,12,13].

Follicle development and sex steroidogenesis are two fundamental roles of the ovary, responsible for oogenesis and endocrine functions, respectively [14]. Sex steroidogenesis in the ovary is involved with a series of enzymes and potentially regulated by various factors; thus, it has attracted much attention in the study of reproductive regulation [15]. Specifically, cholesterol transported to the inner mitochondrial membrane by steroidogenic acute regulatory protein (StAR) is then converted to pregnenolone by P450 side-chain cleavage (P450scc). Pregnenolone is catalyzed by 3β-hydroxysteroid dehydrogenase (3β-HSD) to progesterone, which is hydroxylated by 17α-hydroxylase-17,20-desmolase (P450c17) to androstenedione. Next, androstenedione is transferred to granulosa cells, where it is converted to estrone by cytochrome P450 aromatase (P450arom), then to estradiol via 17β-hydroxysteroid dehydrogenase (17β-HSD) [16]. Gonadotropins play an important regulatory role during sex steroid synthesis, in which luteinizing hormones (LHs) transported into theca cells regulate P450c17, leading to androstenedione synthesis and secretion, while granulosa cells are stimulated by follicle-stimulating hormones (FSHs) to induce P450arom for estrogen production [17]. There have been numerous studies performed on the influence of adiponectin on steroidogenesis. Researchers have found that adiponectin regulates the secretion of progesterone, estradiol, and testosterone in the ovaries by regulating StAR, P450scc, P450c17, P450arom, and 17-hydroxylase [12,18,19]. In addition, adiponectin can also affect the release of GnRH and gonadotropin and the expression of gonadotropin receptors [12,18,19,20].

Wild ground squirrels (*Citellus dauricus* Brandt) are typical seasonal breeding mammals whose reproductive activity exhibits cyclic variations from year to year [21,22,23,24,25,26,27]. During the breeding season (April to May), germ cells mature and wild ground squirrels perform activities, such as mating, giving birth, and rearing litters, which they do not do during the non-breeding season (June to the following March). Many of our previous studies on the physiological properties of seasonal breeding animals have found that both gonads and gonadal appendages of animals in the breeding season appear physiologically active [21,22,23,24,25,26,27]. The ovaries of female ground squirrels are significantly enlarged during the breeding season, with the appearance of Graafian follicles and corpora lutea accompanied by active steroid synthesis and elevated sex hormone concentrations [26,28,29]. It is not completely clear what factors regulate this physiological phenomenon and what role adiponectin plays in it. The goals of this study are to investigate the differences in circulating adiponectin concentrations and steroid synthesis in the ovaries of wild ground squirrels during the breeding and non-breeding seasons and to evaluate the potential association of altered adiponectin concentrations with ovarian morphology, steroidogenesis, and sex hormone production in ovaries of wild ground squirrels.

## 2. Results

### 2.1. Seasonal Changes in Ovarian Tissue Morphology and Sex Hormone Levels

**Figure 1:**
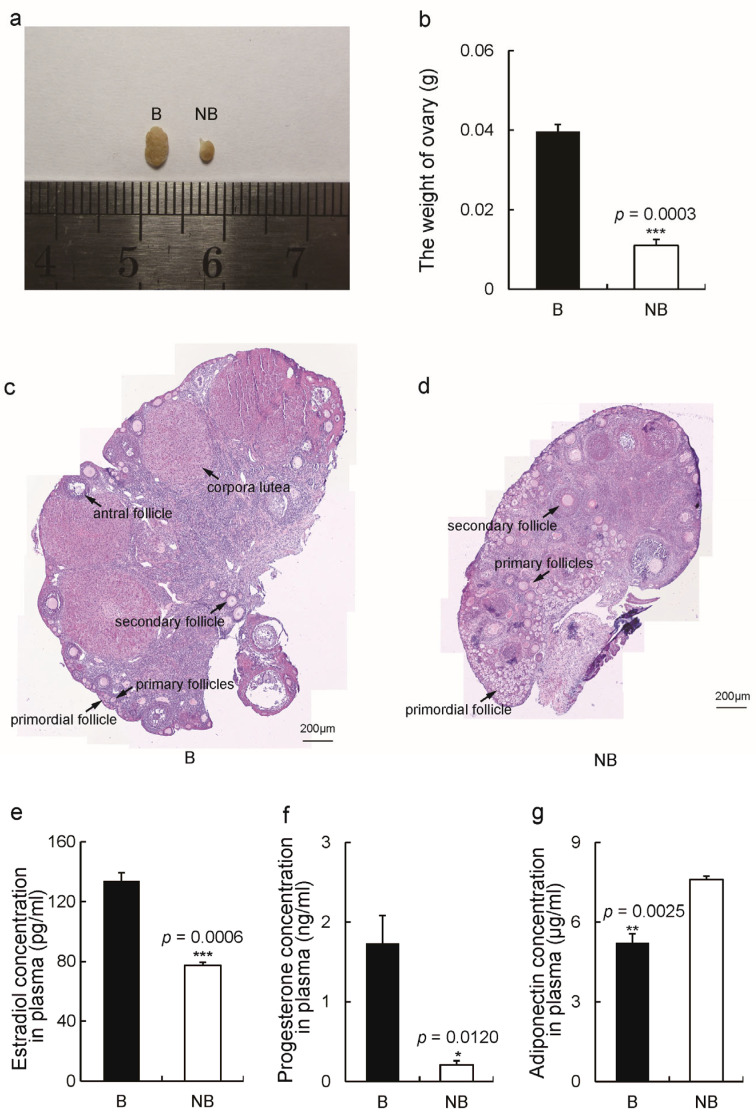
Seasonal changes in the size of the ovaries of wild ground squirrels during the breeding period (**left**) and the non-breeding period (**right**) (**a**). The weight of ovaries during the two periods (**b**). Ovarian follicle composition in wild ground squirrels during the breeding (**c**) and non-breeding (**d**) seasons. The circulating concentration of estradiol (**e**), progesterone (**f**), and adiponectin (**g**) in the plasma of wild ground squirrels during the breeding and non-breeding seasons. Abbreviations: B, the breeding season; NB, the non-breeding season. The error bars represent means ± s.e.m. * represented *p* < 0.05; ** represented *p* < 0.01; *** represented *p* < 0.001. Bar, 200 µm.

The morphological and histological observations of ovaries during the breeding and non-breeding seasons are shown in Figure 1. The volume and weight of ovaries were significantly higher in the breeding season (Figure 1a) than in the non-breeding season (Figure 1b). Follicles of all developmental stages, including primordial, primary, secondary, antral follicles, and corpora lutea, were present in the ovaries during the breeding season (Figure 1c), whereas the ovaries in the non-breeding season had no corpora lutea and antral follicles and a predominance of primordial and primary follicles (Figure 1d). In addition, the plasma levels of estradiol and progesterone were significantly higher in the breeding season than in the non-breeding season, while the circulating adiponectin concentrations were significantly lower in the breeding season (Figure 1e–g).

### 2.2. Expression of Adiponectin and Its Receptors in the Ovaries of Wild Ground Squirrels

**Figure 2:**
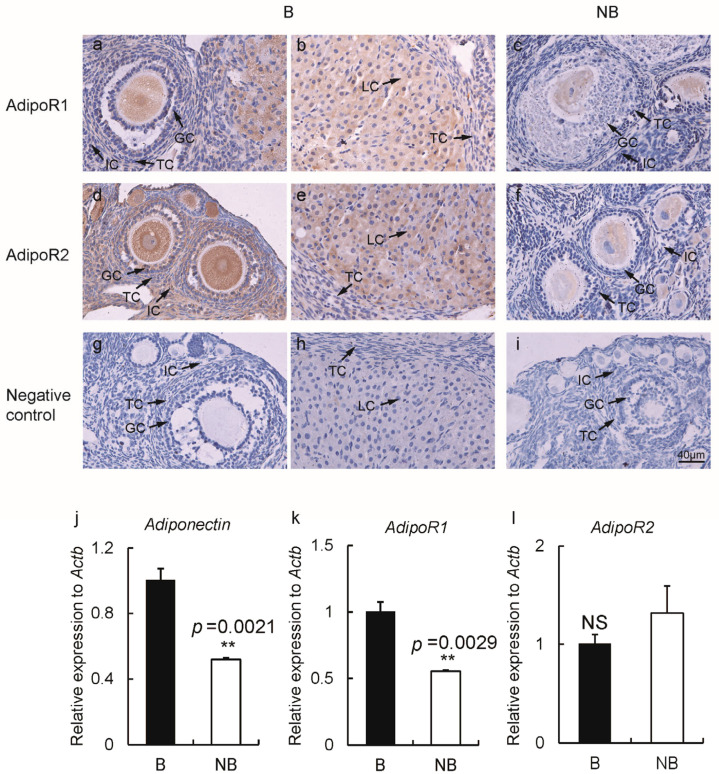
Immunolocalizations of AdipoR1 (**a**–**c**) and AdipoR2 (**d**–**f**) during the breeding season (**first two columns**) and the non-breeding season (**third column**). Negative control of two periods represented in the last row (**g**–**i**). The gene expression levels of *Adiponectin* (**j**), *AdipoR1* (**k**), and *AdipoR2* (**l**) in the breeding and non-breeding periods (*n* = 3 for each group). Abbreviations: B represents the breeding season; NB represents the non-breeding season; GC represents granulosa cells; TC indicates theca cells; IC indicates interstitial cells; LC represents luteal cells. Bar, 40 µm. The error bars represent means ± s.e.m. ** represented *p* < 0.01; NS represents no significance.

Immunolocalization of adiponectin receptors (AdipoR1, AdipoR2) in ovaries showed that AdipoR1 and AdipoR2 were localized in the cytoplasm of theca cells, interstitial cells, luteal cells, and granulosa cells, with strong staining in ovaries during the breeding season (Figure 2a,b,d,e), while little staining was observed during the non-breeding season (Figure 2c,f). There was no signal in the negative control group (Figure 2g–i). The results of immunostainings are summarized in Table 1. Real-time quantitative PCR results indicated that the expression levels of adiponectin and AdipoR1 in ovaries were significantly higher in the breeding season than in the non-breeding season (Figure 2j–l).

### 2.3. Expression of Gonadotropin Receptors and Steroidogenic Enzymes in the Ovaries of Wild Ground Squirrels

**Figure 3:**
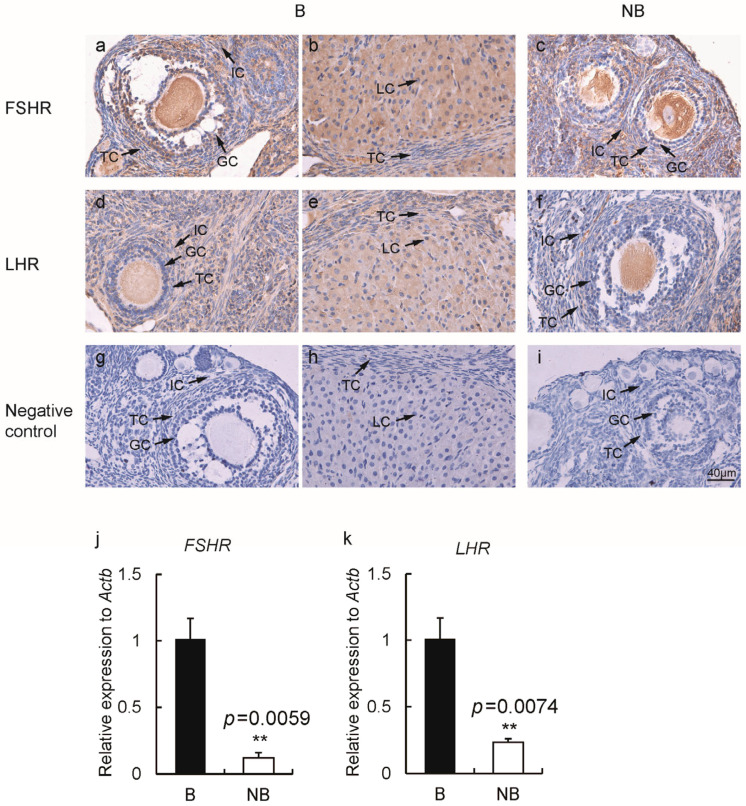
Immunolocalizations of FSHR (**a**–**c**) and LHR (**d**–**f**) during the breeding season (**first two columns**) and the non-breeding season (**third column**). Negative control of two periods represented in the last row (**g**–**i**). The gene expression levels of *Fshr* (**j**) and *Lhr* (**k**) in the breeding and non-breeding periods (*n* = 3 for each group). Abbreviations: B represents the breeding season; NB represents the non-breeding season; GC represents granulosa cells; TC indicates theca cells; IC indicates interstitial cells; LC represents luteal cells. Bar, 40 µm. The error bars represent means ± s.e.m. ** represented *p* < 0.01.

The immunolocalization of gonadotropin receptors (FSHR, LHR) in ovaries was examined, which showed that FSHR (Figure 3a–c) and LHR (Figure 3d–f) were expressed in the cytoplasm of ovarian cells during the breeding and non-breeding seasons, and the expression signals were stronger during the breeding season. There was no signal in the negative control (Figure 3g–i). The results of immunostainings are summarized in Table 2. The expression levels of *Fshr* and *Lhr* were significantly higher in the ovaries during the breeding season than the non-breeding season (Figure 3j,k).

**Figure 4:**
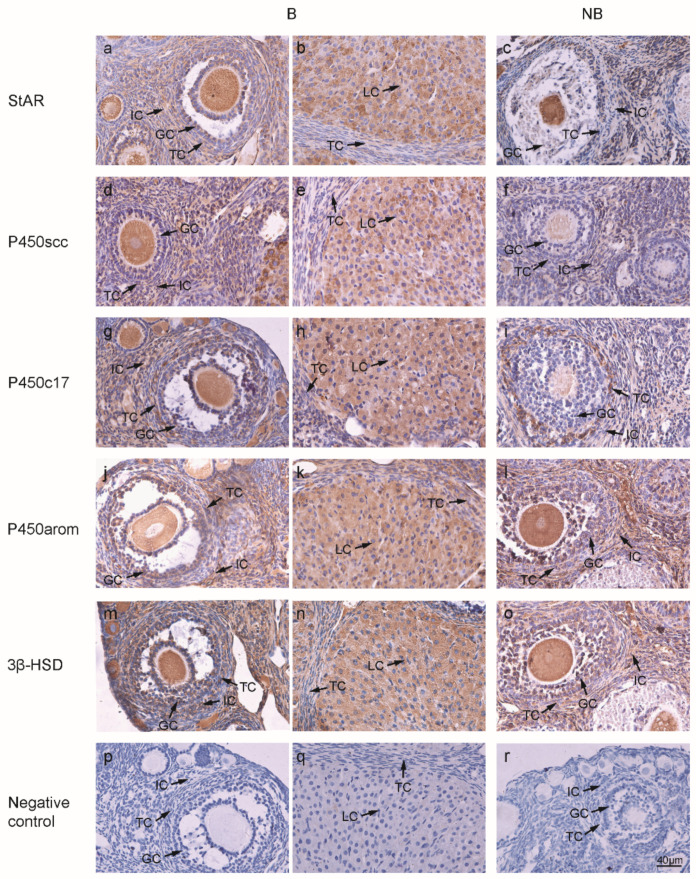
Immunolocalizations of StAR (**a**–**c**), P450scc (**d**–**f**), P450c17 (**g**-**i**), P450arom (**j**–**l**), and 3β-HSD (**m**–**o**) during the breeding (**first two columns**) and non-breeding seasons (**third column**). Negative control of two periods represented in the last row (**p**–**r**). Abbreviations: B represents the breeding season; NB represents the non-breeding season; GC represents granulosa cells; TC indicates theca cells; IC indicates interstitial cells; LC represents luteal cells. Bar, 40 µm.

**Figure 5:**
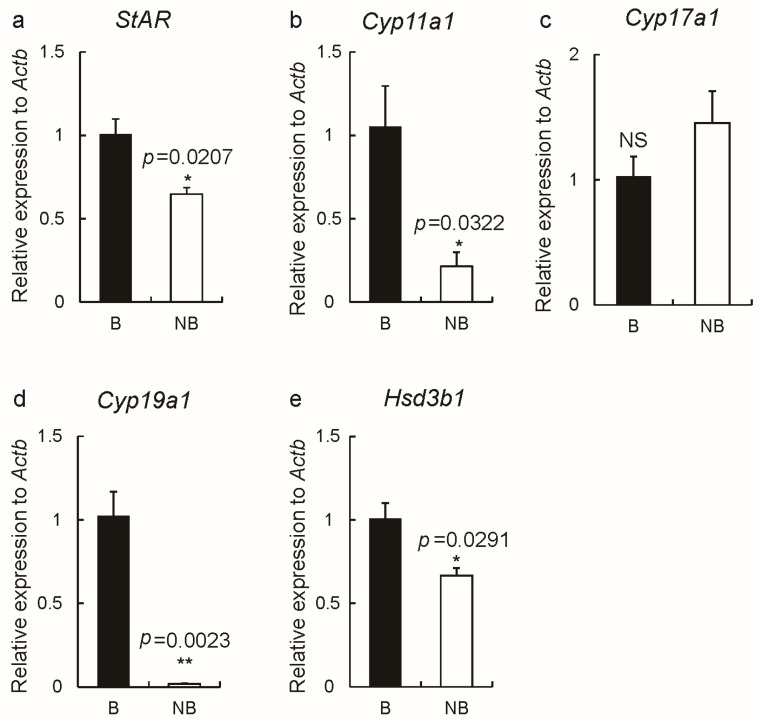
The gene expression levels of *Star* (**a**), *Cyp11a1* (**b**), *Cyp17a1* (**c**), *Cyp19a1* (**d**), and *Hsd3b1* (**e**) in the breeding and non-breeding periods (*n* = 3 for each group). Abbreviations: B represents the breeding season; NB represents the non-breeding season. The error bars represent means ± s.e.m. * represented *p* < 0.05; ** represented *p* < 0.01; NS represents no significance.

Steroidogenic enzymes (StAR, P450scc, P450c17, P450arom, and 3β-HSD) were expressed in ovaries of wild ground squirrels in the breeding and non-breeding seasons, localized to the cytoplasm (Figure 4a–o). There was no signal in the negative control group (Figure 4p–r). The results of immunostainings are summarized in Table 3. Real-time quantitative PCR results showed that the expression levels of *Star*, *Cyp11a1*, *Cyp19a1,* and *Hsd3b1* in ovaries were significantly higher in the breeding season than in the non-breeding season (Figure 5a–e).

### 2.4. Transcriptome Data Analysis in Seasonal Alterations in the Ovary of Wild Ground Squirrels

**Figure 6:**
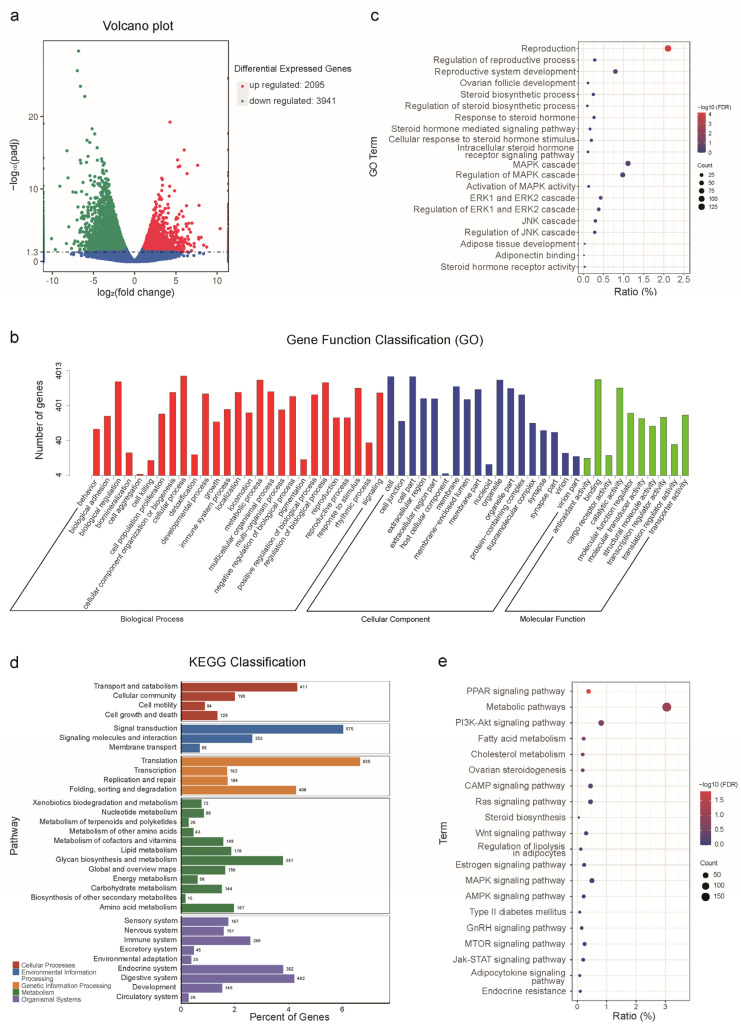
Volcano plot of genes expressed in the ovaries of wild ground squirrels during the breeding and non-breeding seasons (**a**). Functional annotation of DEGs in the ovaries of the two periods based on gene ontology (GO) categorization (**b**). GO significant enrichment bubble diagram of DEGs in the ovaries of the two periods (**c**). KEGG enrichment histogram of DEGs in the two periods (**d**). KEGG significant enrichment bubble diagram of DEGs in the ovaries of the two periods (**e**).

Differences in gene expression in ovarian tissue during the breeding and non-breeding seasons are shown in Figure 6. Thus, 6036 differentially expressed genes (DEGs) were screened, of which 2095 genes were up-regulated and 3941 genes were down-regulated in the breeding season (Figure 6a). Alignment of DEGs with the GO database revealed that a large number of genes was enriched in biological regulation, metabolic process, reproduction, cell, binding, and so on (Figure 6b). The bubble plot shows that GO terms, including regulation of reproductive process, steroid biosynthetic process, MAPK cascade, and adiponectin binding, which were all significantly enriched (Figure 6c). The pathways of DEGs in organisms were further explored by KEGG classification and the genes were classified into five categories: cellular processes, environmental information processing, genetic information processing, metabolism, and organismal systems. Among them, endocrine system, signal transduction, and translation pathways had a large number of genes enriched (Figure 6d). KEGG bubble plots indicated that differentially expressed genes were enriched in ovarian steroidogenesis, adipocytokine signaling pathway, and the mitogen-activated protein kinase (MAPK) signaling pathway (Figure 6e).

### 2.5. Seasonal Changes in MAPK Gene Expression in the Ovaries of Wild Ground Squirrels

**Figure 7:**
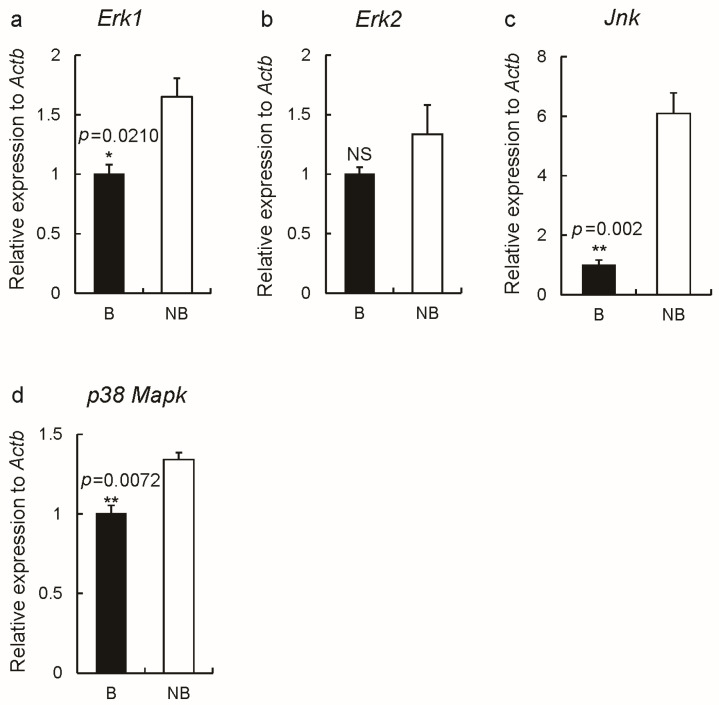
The gene expression levels of *Erk1* (**a**), *Erk2* (**b**), *Jnk* (**c**), and *p38 Mapk* (**d**) in the breeding and non-breeding periods (*n* = 3 for each group). Abbreviations: B represents the breeding season; NB represents the non-breeding season. The error bars represent means ± s.e.m. * represented *p* < 0.05; ** represented *p* < 0.01; NS represents no significance.

The expression levels of MAPK signaling-pathway-related genes (*Erk1*, *Erk2*, *p38 Mapk*, *Jnk*) in the ovarian tissues during the breeding and non-breeding seasons are shown in Figure 7. The results showed that MAPK gene expression was higher in the non-breeding season compared to the breeding season and some genes (*Erk1*, *p38 Mapk*, *Jnk*) (Figure 7a–d) showed significant differences in expression.

## 3. Discussion

This study investigated, for the first time, the relationship between adiponectin and the changes in ovarian function in seasonal breeding animals. Our results showed that ovaries of wild ground squirrels are larger and more active during the breeding season compared to the non-breeding season, with more steroidogenesis, reduced adiponectin secretion, and some altered pathways.

The reproductive organs of seasonal breeding mammals show enlargement and physiological activity during the breeding season [24,26,27,29,30,31,32]. In this study, the morphological results showed that in April, the ovaries of wild ground squirrels became larger in size and heavier in weight. The hematoxylin–eosin (HE) staining results indicated the presence of primordial, primary and secondary follicles, antral follicles, and corpora lutea in the breeding ovaries, whereas in the non-breeding season, the ovaries had only preantral follicles (primordial, primary, and secondary follicles). This illustrates that the ovary is arrested in follicular development during the non-breeding season, at which time, it has no dominant follicle and does not ovulate. Similar changes in ovary morphology have been reported in other seasonal breeding animals, including water voles, golden hamsters, and ferrets [33,34,35]. The ovarian and uterine weights of water voles exhibit significant annual rhythms, with the ovaries being heavier and presenting the maximum number of corpora lutea during the breeding season [33]. Therefore, the ovaries of seasonal breeding mammals show distinct morphological alterations, as evidenced by increased volume, further follicular development, and ovulation during the breeding season, and the results of the present study agree with those of previous studies.

Previous studies have demonstrated that, in addition to adipocytes, adiponectin can be produced and secreted by several other tissues, including skeletal muscle and cardiac muscle, which can play important autocrine and paracrine roles [36]. In our results, adiponectin receptors, AdipoR1 and AdipoR2, were expressed in the ovarian theca, interstitial, luteal, and granulosa cells, suggesting that adiponectin can modulate the function of these cells and may play both autocrine and paracrine roles in ovarian cells. Adiponectin receptors, including AdipoR1, AdipoR2, and T-cadherin, are expressed in various tissues, including adipose tissue, skeletal muscle, and liver [1]. The presence of adiponectin receptors in reproductive organs was also demonstrated, suggesting a regulatory role of adiponectin on the reproductive system [10,11,37]. Once adiponectin binds to AdipoR1 and AdipoR2, a series of downstream signals (mainly including AMP-activated protein kinase (AMPK), p38 mitogen-activated protein kinase (p38 MAPK), etc.) is initiated to exert a series of physiological activities in the body [1]. In addition, steroidogenic enzymes and gonadotropin receptors were also expressed in these cells, implying that adiponectin regulation and steroidogenesis can occur within the same cell.

We observed, via qPCR, that the expressions of steroidogenic enzymes (StAR, P450scc, P450arom, and 3β-HSD) and gonadotropin receptors (FSHR and LHR) in the ovarian cells were significantly increased during the breeding season. The increased expression of StAR, P450scc, and 3β-HSD promoted the conversion of cholesterol to progesterone, whereas P450arom converted androgen to estradiol in response to FSH stimulation. This agrees with the ELISA results, showing significantly higher circulating progesterone and estradiol concentrations during the breeding season. At the same time, the plasma level of adiponectin was significantly lower in the breeding season than in the non-breeding season. However, the expression levels of adiponectin and AdipoR1 in the ovary were significantly higher in the breeding season, according to qPCR, which may be a compensatory mechanism and/or a negative feedback regulation of the receptors by hormones [38,39]. For some hormones, the body compensatively increases their production and receptors when their concentrations decrease [38]. In addition, peptide hormones accelerate receptor invagination and degradation upon binding to the receptor, resulting in a decrease in the amount of receptor [39]. We speculated that the down-regulation of adiponectin in the breeding period might promote the expression of ovarian steroidogenic enzymes, which, in turn, resulted in the up-regulation of hormones. Indeed, the ability of adiponectin to influence steroid synthesis has been demonstrated by numerous studies [12,40,41]. Adiponectin significantly reduced the mRNA expression levels of LHR, CYP11A1, CYP17A1, and the secretion of progesterone and androstenedione in bovine ovarian cells cultured in vitro [12]. In addition, adiponectin inhibited the expression of StAR, CYP11A1, and 3β-HSD in buffalo luteal cells [40]. The expression levels of StAR and CYP11A1 were significantly down-regulated in mouse ovaries after adiponectin treatment [41]. In seasonal breeder Eurasian beavers, the expressions of adiponectin and its receptors were similarly dependent on the cycle of the breeding season and could be linked to reproductive processes through the hypothalamus–pituitary–adrenocortical (HPA) axis [42]. Therefore, the present results found that circulating adiponectin concentration was negatively correlated with the expression levels of steroidogenic enzymes, which could potentially account for seasonal alterations in ovarian function of wild ground squirrels.

Alterations in adiponectin and steroidogenesis are supported by transcriptomic data in the ovaries of wild ground squirrels. After comparing the ovarian transcriptome data between the breeding and non-breeding seasons, we found that many differentially expressed genes were related to the regulation of reproductive development, steroidogenesis, and adiponectin. In addition, we found that the MAPK pathway, the key downstream signal of adiponectin, was enriched by DEGs. The expression of the three major MAPK signaling pathways was examined by qPCR and that of extracellular-signal-regulated kinase 1/2 (ERK1/2), p38 MAPK, and c-Jun N-terminal kinase (JNK) was found to be significantly different between two periods. This suggests that the effect of adiponectin on steroidogenesis may be mediated through the MAPK signaling pathway. It has been reported that MAPK is a major downstream component of adiponectin signaling and adiponectin promotes p38 MAPK, ERK 1/2, and JNK phosphorylation through the adipoRs and APPL1 complex, thereby activating the MAPK signaling pathway and participating in the regulation of physiological activities [43,44]. Numerous studies have shown the effects of the ERK1/2, p38MAPK, and JNK signaling pathways on steroidogenesis [45,46]. MAPK has been reported to exert an important regulatory role on StAR, the rate-limiting enzyme in steroidogenesis, and is also an important pathway for the role of FSH in gonadal target cells [47,48]. In the present study, ERK1, p38 MAPK, and JNK were all significantly down-regulated during the breeding season, which may have contributed to the up-regulation of steroidogenic enzymes in the ovary. Amsterdam suggested that the inhibition of ERK may be an important mechanism to promote gonadotropin-stimulated steroidogenesis [49], whereas others have pointed to ERK as having anti-apoptotic potential [48]. The activation of p38 MAPK was shown to inhibit StAR gene expression and steroidogenesis [50,51]. In addition, both p38 and JNK may be implicated in apoptotic events in primate preovulatory granulosa cells [52]. Although the effects of MAPK signaling pathways on steroidogenesis are complex and require further investigation, our results support views that adiponectin-mediated up-regulation of ERK1, p38 MAPK, and JNK negatively affects the expression of steroidogenic enzymes [49,50,51].

The results of this study demonstrate that seasonal variations in adiponectin may regulate ovarian steroidogenesis under the mediation of the MAPK signaling pathway, thereby affecting seasonal ovarian function in female ground squirrels. The data obtained open up new prospects for studying the role of adiponectin in reproduction and may indicate its role in the control of reproduction in different seasons (for seasonal animals) and under the influence of various factors (feeding behavior, stress, etc.). In the future, we plan to explore the specific mechanism of the potential pathway in adiponectin effects on ovarian function in vitro.

## 4. Materials and Methods

### 4.1. Animals

The wild adult female ground squirrels (weighing 230–419 g) during the breeding season (April, *n* = 10) and the non-breeding season (June, *n* = 10) used in this study were captured in Zhangjiakou City, Hebei Province, China. Animals were anesthetized with 4% isoflurane, weighed, and phlebotomized in hind limb saphenous vein. Blood samples were collected into heparinized tubes and centrifuged at low speed (3000 rpm, 20 min). We collected the supernatant (plasma) after centrifugation and cryopreserved (−20 °C). The ground squirrels were euthanized using an overdose of pentobarbital (BioDee Co., Beijing, China), whose ovaries were dissected. The cross-sectional diameter of the ovaries (*n* = 10) was measured using a straightedge and the ovaries (*n* = 10) were weighed using an electronic balance. Ovaries from one side of wild ground squirrels (*n* = 10) were fixed in 4% paraformaldehyde for histological and immunohistochemical analysis, while ovaries from the other side (*n* = 10) were immediately frozen in liquid nitrogen and stored at −80 °C for gene expression analysis. All animal test procedures were in accordance with the Policy on the Care and Use of Animals of the Ethics Committee of Beijing Forestry University (EAWC_BJFU_202008) and approved by the Hebei Provincial Department of Agriculture (JNZF11/2007).

### 4.2. Histology

Ovarian tissues soaked in 4% paraformaldehyde for 24 h were removed, rinsed in running water for one day, dehydrated in an alcohol–xylene gradient, and embedded with paraffin. The paraffin blocks were sectioned at 5 μm. Paraffin wafers are spread out on warm water and picked up with an adhesive slide so that the wafers with tissue are attached to the surface of the slide. The deparaffinized tissue sections were stained with hematoxylin–eosin. The stained sections were gradient dehydrated and sealed with a coverslip glued on with neutral balsam. The prepared tissue sections were observed under the microscope for overall tissue morphology, follicle distribution, and cell type.

### 4.3. Immunohistochemistry

Deparaffinized ovarian sections were incubated in citric-acid-buffered solution and subsequently blocked with 10% goat serum. Tissues were incubated overnight (4 °C) with primary antibodies including AdipoR1 (sc-46748, Santa Cruz Biotechnology, Santa Cruz, CA, USA), AdipoR2 (sc-46751, Santa Cruz Biotechnology, Santa Cruz, CA, USA), FSHR (bs-20658R, Bioss Biotechnology, Beijing, China), LHR (bs-6431R, Bioss Biotechnology, Beijing, China), StAR (bs-20387R, Bioss Biotechnology, Beijing, China), P450scc (bs-10099R, Bioss Biotechnology, Beijing, China), P450c17 (bs-3853R, Bioss Biotechnology, Beijing, China), P450arom (ab18995, Abcam, Shanghai, China), HSD3B1 (bs-3906R, Bioss Biotechnology, Beijing, China), and control group with normal rabbit IgG instead of the primary antibody. The specificity of the AdipoR1, AdipoR2, FSHR, LHR, StAR, P450scc, P450c17, P450arom, and 3β-HSD antibodies have been described in previous studies [23,53,54]. Tissues were further processed with a goat anti-rabbit IgG/HRP Kit (bs-0295g-hrp, BIOSs biotechnology, Beijing, China), stained by diaminobenzidine solution (30 mg DAB, 150 mL 0.05 M pH 7.6 Tris HCl solution, 25 µL H_2_O_2_), and counterstained with hematoxylin. We scored the positive signals of DAB staining of ovarian tissue with ImageJ (1.53k, National Institutes of Health, Bethesda, MD, USA) (− represents negative, +++ represents strong positive) [23,55].

### 4.4. Hormone Measurement

The concentrations of estradiol and progesterone in the plasma of wild ground squirrels were measured using ELISA kits (CSB-E05110r for estradiol, CSB-E07282r for progesterone, CSB-E07271r for adiponectin, Cusabio Biotech Co., Ltd., Wuhan, China) according to the protocol. The data at 450 nm were subsequently read with a microplate reader (PT 3502 g, Beijing Potenov Technology Co., Ltd., Beijing, China). The intra-assay and inter-assay coefficient of variation for estradiol were 5.1% and 8.9%, respectively. In the hormone determination of progesterone, the intra- and inter-assay co-efficient of variation were 3.9% and 6.7%, respectively. The intra-assay co-efficient of variation was 4.5% and the inter-assay co-efficient of variation was 7.3% in the hormone determination of adiponectin.

### 4.5. Real-Time Quantitative PCR

Total RNA from the ovaries of wild ground squirrels was extracted using the Trizol Kit (Invitrogen, Carlsbad, CA, USA) and the concentration was adjusted to 250 ng/µL. cDNA was synthesized with StarScript II RT MasterMix, RNA (1000 ng), and random primer (GenStar, Beijing, China). The 10 µL system (3 μL cDNA, 0.3 μL of forward and reverse primers (100 μg/mL), 5 μL 2× Power SYBR Green PCR master mix, and 1.4 μL ddH2O) was configured according to the FastStart DNA MasterPlast SYBR Green Kit (Roche Molecular Systems Inc., Basel, Switzerland) protocol and the primer design is shown in Table 4. The data were measured by an ABI PRISM 7500 Fast Real-Time PCR System (Applied Biosystems, Foster City, CA, USA). A 95 °C preheating for 10 min was followed by 40 cycles (95 °C for 30 s, 60 °C for 30 s, 72 °C for 30 s) and finally a 60–95 °C melt curve progression. The relative expression of target genes was analyzed according to the expression of internal reference *β-actin*.

### 4.6. Construction of Transcriptome Libraries

Total RNA (1000 ng) of ovarian tissue (*n* = 3) was extracted following the protocol of Trizol kit. The mRNAs in the total RNA were enriched by magnetic beads with oligo (DT) and subsequently randomly interrupted. The fragmented mRNA was synthesized as cDNA single stranded using the M-MuLV reverse transcriptase system, followed by the addition of DNA polymerase I, dNTPs, and buffer to synthesize stable cDNA double strands. cDNA was purified by the AMPure XP system (Beckman Coulter, Beverly, MA, USA) and 200–250 bp cDNA was selected for PCR amplification. The amplification products were purified again using AMPure XP Beads, resulting in a cDNA library. Transcriptome libraries were sequenced by the Illumina HiSeq platform (Allwegene, Beijing, China).

### 4.7. Transcriptome Data Analysis

The clean reads were obtained by quality control of raw reads. Quantification of gene expression levels was calculated according to the method in Xie’s article [55]. The data were aligned using DESeq2 (DESeq2_1.20.0) software and significant differentially expressed genes (*p* value < 0.05) were selected. GO functional classification of differentially expressed genes was performed using the blast2GO algorithm and the KEGG mapping analysis of genes was performed by the KEGG pathway alignment analysis tool to obtain functional annotation as well as pathway enrichment analysis results.

### 4.8. Statistical Analysis

GraphPad Prism 6 software was used to analyze the significance of differences by Student’s *t* test. *p* < 0.05 was set as the indicator of statistical significance.

## Figures and Tables

**Table 1:** Relative abundance of target proteins of AdipoR1 and AdipoR2 in the ovaries of wild ground squirrels during the breeding and non-breeding seasons.

Antibodies	Granulosa Cells		Theca Cells		Interstitial Cells		Luteal Cells	
	B	NB	B	NB	B	NB	B	NB
AdipoR1	++	+	++	+	++	+	+++	/
AdipoR2	+++	+	++	+	++	+	+++	/

Note: B indicates breeding season; NB indicates non-breeding season. /, no data; +, low positive staining; ++, positive staining; +++, strong positive staining.

**Table 2:** Relative abundance of target proteins of FSHR and LHR in the ovaries of wild ground squirrels during the breeding and non-breeding seasons.

Antibodies	Granulosa Cells		Theca Cells		Interstitial Cells		Luteal Cells	
	B	NB	B	NB	B	NB	B	NB
FSHR	+++	++	+++	++	+++	++	+++	/
LHR	+++	+	+++	++	+++	+	+++	/

Note: B indicates breeding season; NB indicates non-breeding season. /, no data; +, low positive staining; ++, positive staining; +++, strong positive staining.

**Table 3:** Relative abundance of target proteins of StAR, P450scc, P450c17, P450arom, and 3β-HSD in the ovaries of wild ground squirrels during the breeding and non-breeding seasons.

Antibodies	Granulosa Cells		Theca Cells		Interstitial Cells		Luteal Cells	
	B	NB	B	NB	B	NB	B	NB
StAR	+++	+	+++	++	+++	++	+++	/
P450scc	+++	+	+++	++	+++	++	+++	/
P450c17	+++	++	+++	+++	+++	++	+++	/
P450arom	+++	+++	++	++	+++	+++	+++	/
3β-HSD	+++	+++	+++	+++	+++	+++	+++	/

Note: B indicates breeding season; NB indicates non-breeding season. /, no data; +, low positive staining; ++, positive staining; +++, strong positive staining.

**Table 4:** Oligonucleotide sequences for real-time quantitative PCR.

Gene Symbol	Primer Forward (5′-3′)	Primer Reverse (5′-3′)
*Adiponectin*	GCGGGTCTTGTTGGTCCTAA	CACACTGAAGGCTGATCGGT
*AdipoR1*	GTTCCTGGGACTTGGCTTGA	CAGGAAAGAAGCGCTCAGGA
*AdipoR2*	GACGGGCAACATTTGGACAC	AAAGGCAGAGAATGGCTCCC
*Star*	TTGGGCATACTCAACAACCA	CCTTGACATTTGGGTTCCAC
*Cyp11a1*	ACATGGCCAAGATGGTCCAG	TTCTCGACCCATGGCATAGC
*Cyp17a1*	TTTTGGCCCAAGTCAAAGAC	CCAGCTGATAGTGACCGACA
*Cyp19a1*	ATTTGGCAGCAAACTTGGGT	CAGTCTGTCCAGGTGCCTTA
*Fshr*	CGTCATGGTATTGGGCTGGA	GACCACAAAGGCCAGGACAT
*Lhr*	GCCTCGCCAGACTATCTCTC	GGGTTCGGATGCCTGTGTTA
*Hsd3b1*	GTTCCTGGGACTTGGCTTGA	CAGGAAAGAAGCGCTCAGGA
*ERK1*	TATCAACACCACCTGCGACC	GCCCACAGACCAGATGTCAA
*ERK2*	CCCATTGCTGAAGCACCATT	AGTGGGTAAGCTGAGACGGG
*P38 MAPK*	TCGGCACACTGATGACGAAA	TCATGGCTTGGCATCCTGTT
*JNK*	CCTGTAGGTCCTGGCATTGG	AGTAGGCTTTGGGACCTCCT
*β* *-actin*	GACTCGTCGTACTCCTGCTT	AAGACCTCTATGCCAACACC

## Data Availability

Not applicable.
